# Phenotypic and genotypic within-host diversity of *Pseudomonas aeruginosa* urinary isolates

**DOI:** 10.1038/s41598-022-09234-5

**Published:** 2022-03-30

**Authors:** Agnès Cottalorda, Sandrine Dahyot, Anaïs Soares, Kevin Alexandre, Isabelle Zorgniotti, Manuel Etienne, Estelle Jumas-Bilak, Martine Pestel-Caron

**Affiliations:** 1 GRAM 2.0, Normandie Univ, UNIROUEN, UNICAEN, 76000 Rouen, France; 2 GRAM 2.0, CHU Rouen, Department of Microbiology, Normandie Univ, UNIROUEN, UNICAEN, 76000 Rouen, France; 3 GRAM 2.0, CHU Rouen, Department of Infectious Diseases, Normandie Univ, UNIROUEN, UNICAEN, 76000 Rouen, France; 4 Team Pathogènes Hydriques Santé Environnement, UMR 5569 HydroSciences Montpellier, University of Montpellier, Montpellier, France

**Keywords:** Clinical microbiology, Urological manifestations

## Abstract

This study aimed to assess phenotypic and molecular inter-patient and within-host diversity of *Pseudomonas aeruginosa* isolates responsible for urinary tract infection (UTI) or asymptomatic bacteriuria (AB). Clinical data of 120 consecutive *P. aeruginosa* UTI (*n* = 40) and AB (*n* = 80) were prospectively analyzed. Up to five *P. aeruginosa* isolates per sample were collected. Antimicrobial susceptibility testing (AST) was determined for all isolates (*n* = 591); a subset of 358 was characterized by multilocus sequence typing. 444 isolates (75%) were non-multidrug resistant (MDR), 113 (19%) were MDR, and 34 (6%) were extensively drug resistant. A genetically highly diverse population was observed (64 sequence types [STs]), without strict correlation between genotypes and clinical settings. 35 patients (28%; 12 UTIs and 23 ABs) presented distinct antimicrobial resistance (AMR) profiles within a given urine sample, significantly associated with previous carbapenem and fluroquinolones exposure; five of them also exhibited polyclonal UTI or AB (with isolates belonging to two STs). *P. aeruginosa* urinary isolates of these 120 patients were highly diverse, in terms of AMR as well as genetic background. Both within-host AMR and molecular diversity can complicate AST, treatment and control of *P. aeruginosa* UTI.

## Introduction

*Pseudomonas aeruginosa* is an opportunistic pathogen responsible for 7–10% of healthcare-associated urinary tract infections (UTI)^1–3^. *P. aeruginosa* UTI commonly affects patients with underlying conditions, such as urinary tract abnormalities^2,4^, and are particularly promoted by urinary indwelling catheters^2^.

Because of its ability to develop resistance to multiple classes of antibiotics, multidrug resistance (MDR) is frequently reported in *P. aeruginosa* urinary strains^4,5^. This phenomenon, associated with *P. aeruginosa* ability to form biofilm, leads to infections that are difficult to treat^6^.

To explore the diffusion of resistant strains and clonal relatedness of *P. aeruginosa* isolates, a large variety of genotyping methods has been developed such as Multilocus sequence typing (MLST). MLST has successfully identified epidemic high-risk clones resistant to antibiotics and responsible for healthcare-associated infections^7^. On the other hand, within-host diversity of *P. aeruginosa* isolates has been identified in cystic fibrosis (CF) infections, which may complicate patient’s care^8–10^. In contrast, few studies have explored the genetic diversity of *P. aeruginosa* urinary strains; they often included a limited number of strains and mainly focused on multiresistant ones^11,12^. Thus, little is known about both molecular epidemiology and within-sample diversity of *P. aeruginosa* urinary isolates.

In this context, the aims of this study were (1) to investigate antimicrobial resistance (AMR) and genetic background of *P. aeruginosa* isolates collected from UTI or asymptomatic bacteriuria (AB), and (2) to explore within-host diversity of these isolates.

## Results

### Demographic and clinical characteristics of patients

The demographic and clinical characteristics of the 120 consecutive patients suffering from *P. aeruginosa* bacteriuria considered as UTI or AB are presented in Table S1. The mean age of the patients was 64 years, ranging from 1 month to 101 years. 64% (77/120) were male. Mean Charlson index was 5.4. Patients were mostly hospitalized in medical (46/120, 38%) or in surgery (32/120, 27%) wards. Within the 6 months before inclusion, 86% were hospitalized, 68% underwent urinary tract manipulation, and 78% received at least one antibiotic treatment (Table S1). The antibiotic classes previously received by patients were mostly penicillins (72/120, 60%), and cephalosporins (43/120, 36%) (Table S2).

Compared to AB, demographic or clinical characteristics significantly associated with UTI were male sex (78% versus 58%), bacteraemia (30% versus 3%), and uncomplicated diabetes (32% versus 13%) (*P* < 0.05) (Table S1).

### Phenotypic and molecular characteristics of isolates

#### Antimicrobial susceptibility testing (AST)

**Figure 1:**
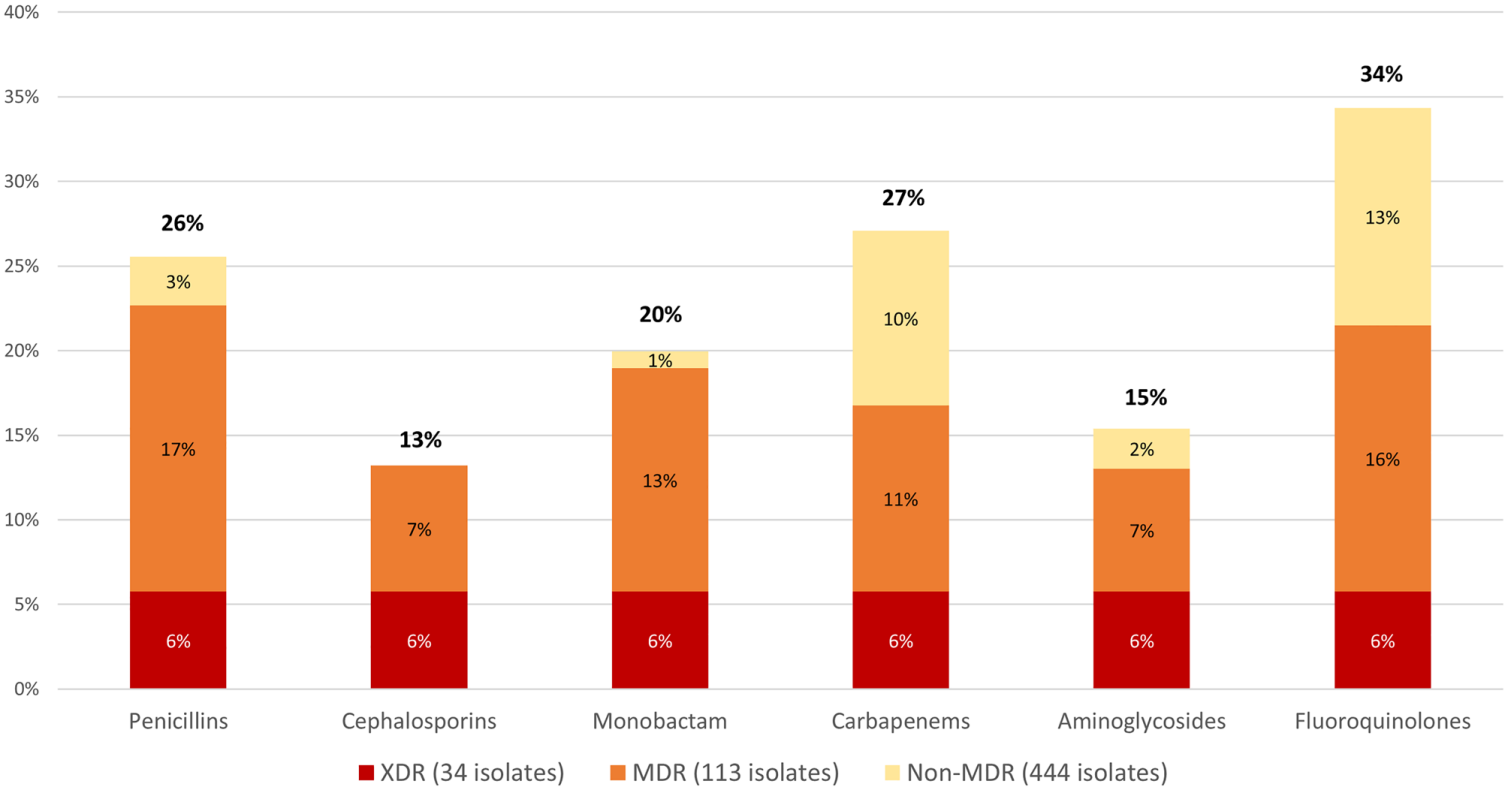
Overall resistance rates of the 591 *P. aeruginosa* urinary isolates to antimicrobial groups. Overall resistance rates are presented for each antimicrobial group in bold, above the bar chart. Percentage of non-MDR (MultiDrug Resistant), MDR, and XDR (eXtensively Drug Resistant) profiles are indicated for each antimicrobial group inside the bar chart.

Among the 591 isolates tested (up to five per urine sample), resistance rates ranged from 13 to 34% depending on the antimicrobial groups (Fig. 1). Low rates (≤ 20%) were observed for cephalosporins, monobactam, and aminoglycosides while rates were higher (≥ 25%) for fluoroquinolones (34%), carbapenems (27%) and penicillins (26%). 444 isolates (75%) were non-MDR (including 318 wild-type isolates [54%]), 113 (19%) were MDR and 34 (6%) were extensively drug resistant (XDR) (Fig. 1).

MDR and/or XDR profiles were significantly more observed for patients with previous urine culture positive for *P. aeruginosa* or antibiotic treatment (especially cephalosporins and fluoroquinolones) (*P* < 0.05) (Table S2). In addition, patients with resistant isolates received a higher mean number of different antimicrobial groups in the past 6 months (i.e. 1.5, 2.6, 3.3 for patients with non-MDR, MDR, and XDR isolates, respectively).

#### Genotyping by MLST

64 STs were identified for the 358 isolates tested (two to three per urine sample). 46 STs were singletons, as identified in only one patient (Table S3). The most predominant STs were ST395 and ST308 (11 patients each), followed by ST253 (7 patients), ST309 and ST235 (6 patients each).

**Figure 2:**
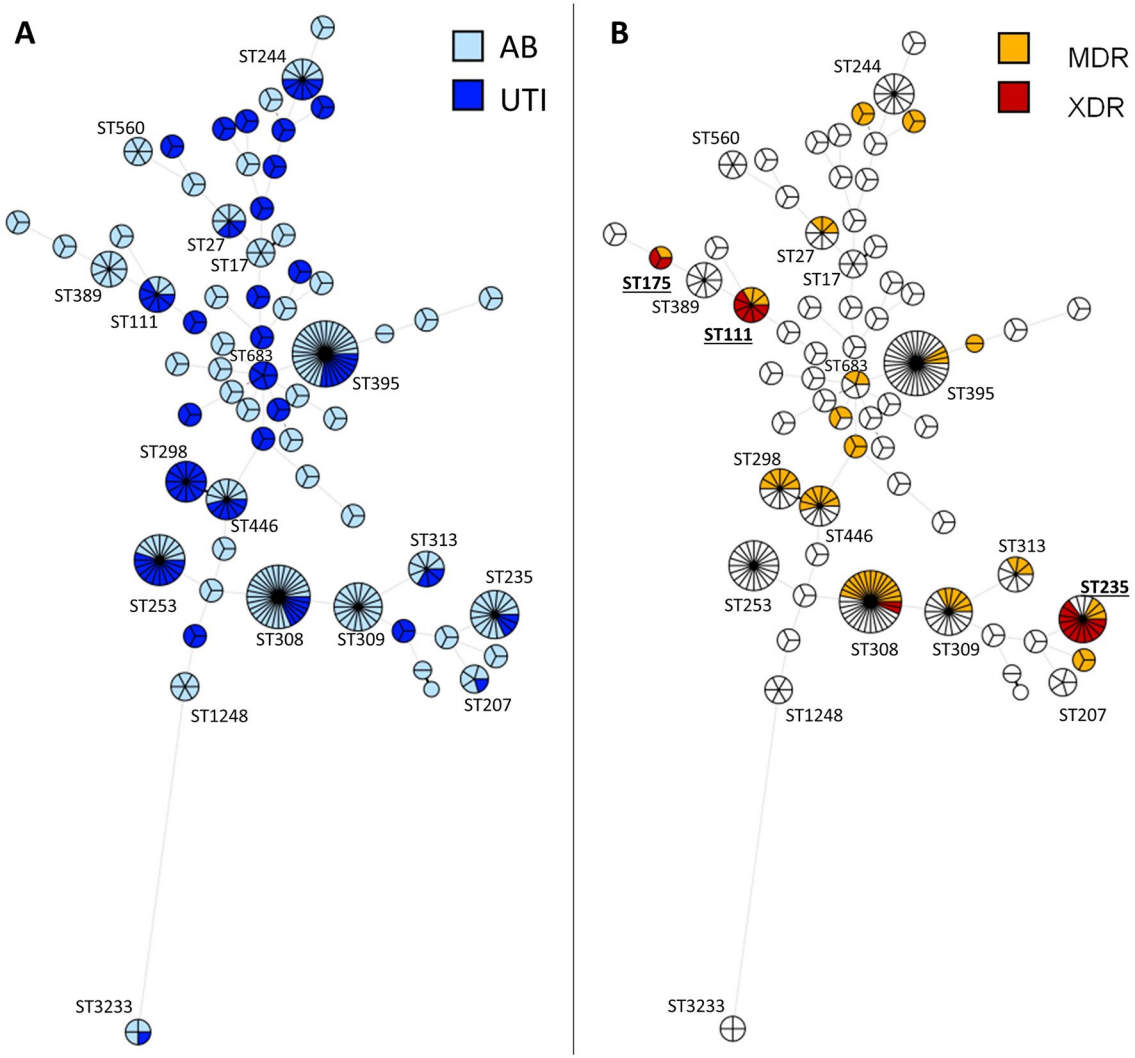
Minimum spanning tree of the 358 *P. aeruginosa* urinary isolates typed by multilocus sequence typing (MLST). The sequences were concatenated and analyzed with BioNumerics. Clustering of MLST profiles was done using a categorical coefficient. The colors used are based (**A**) on clinical contexts (AB: Asymptomatic Bacteriuria; UTI: Urinary Tract Infection) and (**B**) on antimicrobial resistance profiles (MDR: MultiDrug Resistant; XDR: eXtensively Drug Resistant). Each circle represents a sequence type (ST) and its size is proportional to the number of isolates. Length of the lines represent the genetic distance between isolates. Thick, short lines connecting two types denote types differing in a single locus; thin, longer lines connect double-locus variants; and dashed lines indicate the most likely connection between two types differing in more than two loci. STs identified in at least two patients are annotated, and the three most prevalent worldwide epidemic high-risk clones^7^ (**B**) are in bold.

29 STs were identified for the 120 UTI isolates, and 46 STs for the 238 AB isolates. No strict correlation was found between STs and clinical setting, as 11 of the 18 STs identified in at least two patients were observed in both UTI and AB contexts (Fig. 2A). Despite this, ST309 (6 patients) and ST298 (4 patients) tended to be associated with AB and UTI, respectively. Furthermore, 4 STs (ST308, ST235, ST111 and ST175) were mainly associated with MDR and XDR profiles (Fig. 2B).

### Within-host diversity

**Figure 3:**
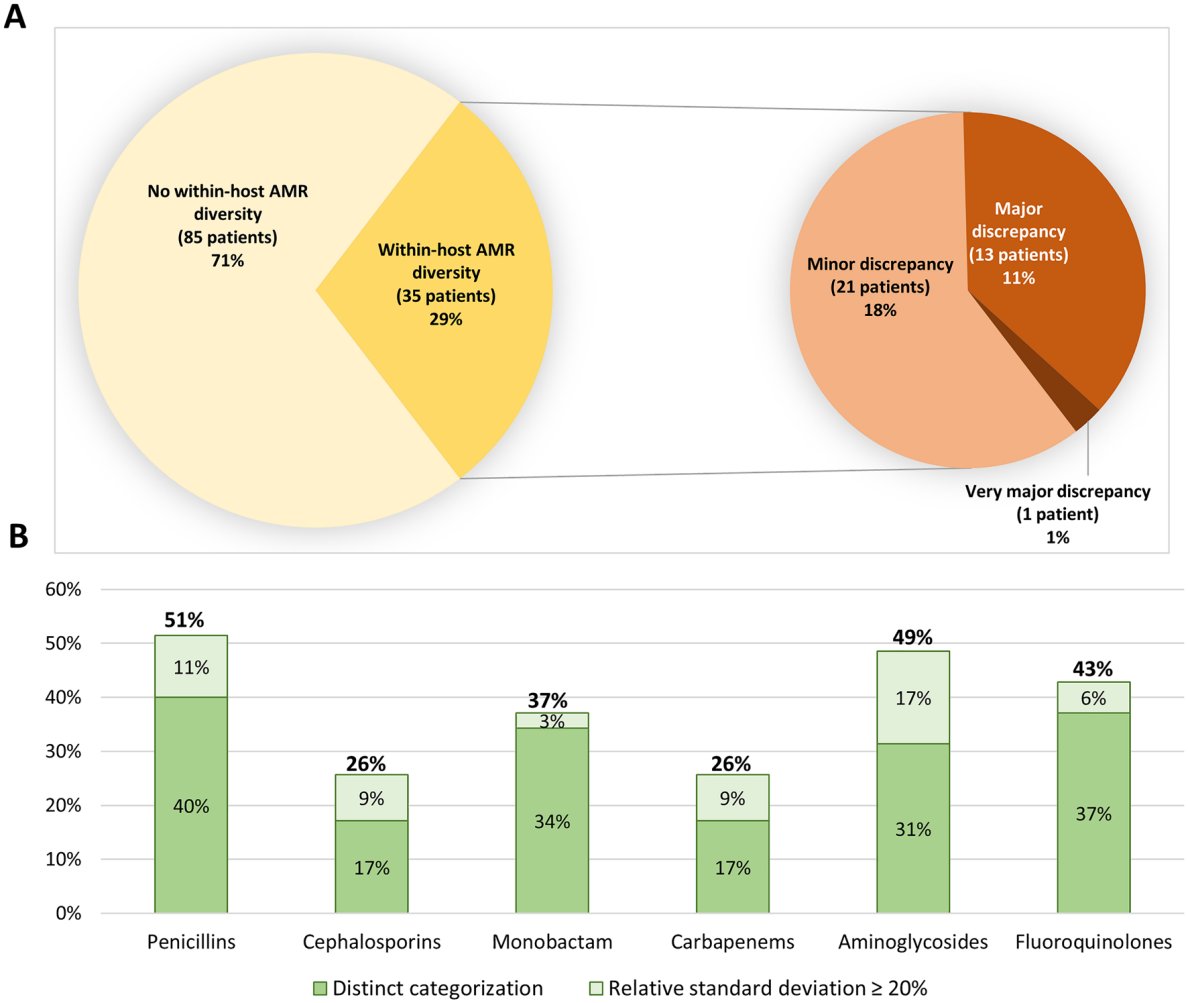
Within-host AMR diversity. (**A**) Number and percent of patients with within-host antimicrobial resistance (AMR) diversity, including minor, major, or very major discrepancies. (**B**) Percent of patients with within-host AMR diversity according to antibiotic classes. Overall diversity rates are presented for each antimicrobial group in bold, above the bar chart. Percentage of AMR diversity due to distinct categorization (susceptible, intermediate or resistant) or relative standard deviation ≥ 20% of the inhibition zone diameters are indicated for each antimicrobial group inside the bar chart.

Within-host diversity was assessed by comparing AST profiles and STs between isolates from a given sample. Among the 120 patients, 85 (71%) presented no within-host AMR diversity (71 with non-MDR, 10 with MDR, and 4 with XDR isolates), whereas 35 presented within-host AMR diversity (Fig. 3A). Minor discrepancies were observed for 21 of them. Interestingly, 14 patients (12%) harbored isolates with two distinct AMR profiles, corresponding to 13 major discrepancies (9 non-MDR/MDR, and 4 MDR/XDR) and one very major (non-MDR/XDR) (Fig. 3A). AMR diversity concerned all antibiotic classes (Fig. 3B). Comparison of clinical characteristics between patients with or without AMR diversity revealed that previous exposure to carbapenems or fluoroquinolones was significantly more frequently observed in patients with AMR diversity (*P* < 0.05) (Table 1).

**Table 1:** Clinical characteristics of patients with phenotypic and molecular within-host diversity.

Characteristics	No diversity (*n* = 85)	AMR diversity (*n* = 35)	AMR diversity associated with genetic diversity (*n* = 5)
**Demographics no. (%)**			
Age (years) [min; max]	64 [0; 97]	64 [1; 101]	54 [36; 83]
Male gender	55 (65%)	22 (63%)	4
**Clinical features no. (%)**			
Urinary tract infection	28 (33%)	12 (34%)	2
Urinary tract catheter	42 (49%)	18 (51%)	3
Bacteraemia caused by *P. aeruginosa*	8/43 (19%)	1/17 (6%)	0/2
**Comorbid diseases no. (%)**			
Urinary comorbidity	40 (47%)	21 (60%)	3
Charlson index (avg ± SD)*	5.4 ± 3.0	5.4 ± 2.5	3.2 ± 2.2
Other comorbidity	9 (11%)	3 (9%)	0
**Clinical histories within the previous 6 months no. (%)**			
Previous urine culture positive to *P. aeruginosa*	8 (9%)	7 (20%)	2
Previous antibiotic treatment	65 (77%)	28 (80%)	4
Penicillins	50 (59%)	22 (63%)	1
Cephalosporins	31 (36%)	12 (34%)	1
Carbapenems	5 (6%)	7 (20%)**	2
Aminoglycosides	17 (20%)	8 (23%)	0
Quinolones	11 (13%)	11 (31%)**	1
Other antimicrobials	35 (41%)	17 (49%)	2
Previous hospitalization	73 (86%)	30 (86%)	5
Previous urinary tract manipulation	59 (69%)	23 (66%)	3
Including previous urological surgery	17 (20%)	4 (11%)	1

*MDR* multidrug resistant, *XDR* extensively drug resistant, *AMR* antimicrobial resistance, *min* minimum, *max* maximum, *SD* standard deviation.

*Charslon index was measured only for adult cases (i.e. 114 patients, 37 with UTI, and 77 with AB).

**P value < 0.05.

Remarkably, urine sample of five patients (4%; 2 with UTI and 3 with AB) harbored isolates with two distinct STs. These STs differed for 4 to 7 alleles for 4 patients and for a single *locus* (*mutL*) for one patient (Table 2). Within-host genetic diversity was always associated with within-host AMR diversity, with either minor (two patients), major (two patients), or very major discrepancies (one patient). With the limit of the small number of patients, this genetic diversity seemed to occur preferentially in patients with prior hospitalization (all the five patients) and previous antibiotic treatment (four patients) (Table 1).

**Table 2:** Allelic profiles of *P. aeruginosa* isolates from patients with polyclonal urinary tract infection or asymptomatic bacteriuria.

Patients	Clinical context	STs	Nb of isolates	*acsA*	*aroE*	*guaA*	*mutL*	*nuoD*	*ppsA*	*trpE*	Nb of alleles differentiating STs
A	UTI	253	2	4	4	16	12	1	6	3	4
		207	1	**47**	4	**5**	**33**	1	6	**40**	
B	AB	2406	2	29	8	36	67	1	6	3	1
		3232	1	29	8	36	**228**	1	6	3	
C	AB	308	2	13	4	5	5	12	7	15	5
		207	1	**47**	4	5	**33**	**1**	**6**	**40**	
D	UTI	683	2	6	5	11	3	4	4	1	7
		3233	1	**87**	**34**	**114**	**37**	**86**	**91**	**170**	
E	AB	483	2	116	5	6	5	3	12	68	7
		446	1	**18**	**4**	**5**	**3**	**1**	**17**	**13**	

The seven alleles (*acsA*, *aroE*, *guaA*, *mutL*, *nuoD*, *ppsA* and *trpE*) that defined sequence type (ST) are indicated here. Alleles that differ between the 2 STs of the isolates of a given patient are in bold.

## Discussion

*P. aeruginosa* is frequently responsible for healthcare-associated UTI, but little is known about the genetic background of the urinary strains or within-host diversity. Thus, we investigated phenotypic and molecular diversity of *P. aeruginosa* in the setting of UTI or AB, by exploring multiple isolates per urine sample.

Patients enrolled in this study presented characteristics previously described as risk factors for *P. aeruginosa* UTI (i.e. male sex, urological disorder, urinary tract catheter and previous UTI/antibiotic therapy/hospitalization)^1,2,4^. The rate of MDR/XDR (25%) isolates was close to that reported worldwide (15–30%)^7^ and to that observed in studies based on *P. aeruginosa* urinary isolates (10.2–41.2%)^4,13^.

Genetic diversity of our isolates was assessed by MLST, commonly used to describe the global epidemiology of *P. aeruginosa*^7,14,15^. A genetically highly diverse population (64 STs) was observed, as previously described for other infections^16^, with no strict correlation between genotype and clinical setting (AB or UTI). Although most of the STs were identified in only one patient, 13 of the 18 spread clones reviewed by Oliver et al.^7^ were identified, including the two predominant clones in our population (ST395, and ST308). Some of them were mainly associated with multiresistance, including the three most predominant worldwide epidemic high-risk clones (ST111, ST175, ST235)^7^. Of note, ST234 and ST235, previously described for *P. aeruginosa* urinary isolates^11,12^, were identified for some of our isolates.

Within-host diversity was then assessed by comparing phenotypes and genotypes of isolates collected from a given sample.

It first revealed that almost one-third of our patients exhibited within-sample AMR diversity that could be associated with phenotypic plasticity. This observation is a major concern because it can lead to treatment failure. This result supports the recommendation to pool several distinguishable colonies of *P. aeruginosa* when performing AST^17^, or even to carry out a direct AST by disk diffusion from urine specimen^18^, in order to identify AMR diversity that could impact the choice of antibiotic treatment. This latter method provides a rapid solution for determining AST and facilitates identification of resistant subpopulations^18^. The AMR diversity was previously observed by Mowat et al.^9^ who analyzed sets of 40 *P. aeruginosa* cystic fibrosis (CF) isolates per sputum sample. Despite the lower number of isolates per sample explored here, the high AMR diversity rate suggests that the urinary tract could be a complex environment that favor adaptation, even if the selective pressure in the urinary tract is different than during chronic long-term CF infections.

Then, our study is the first to demonstrate that *P. aeruginosa* UTI or AB can be polyclonal, as two distinct STs were identified within a urinary sample for five patients (4%). For four of them, at least four alleles distinguished the two clones. Analysis of previous urine samples could have been of interest to establish whether these clones already coexisted, especially for the two patients who had a history of *P. aeruginosa* bacteriuria. Moreover, these five patients were previously hospitalized, and four of them received antimicrobial treatments that could have selected resistant strains. This is supported by the fact that genetic diversity was always correlated with AMR diversity.

Nevertheless, the number of patients harboring polyclonal bacteriuria was smaller than that described by Waine et al.^10^ in the CF context, where 2 to 3 STs were identified for half of the 60 patients included. However, that study was based on a much larger number of isolates (from 27 to 46 per sputum). Further studies with many more isolates per urine sample are therefore needed to evaluate within-sample diversity to a greater extent, and to identify the impact of polyclonal UTI on patient outcome.

On the other hand, the resolution of MLST is limited due to the use of a small number of genes. It would be interesting to further characterize isolates with the same ST by whole genome sequencing given the high discriminatory power of this technology^19,20^. In this way, genomic comparisons of *P. aeruginosa* strains isolated from CF^21^ and non-CF patients^22^ have showed that a parental strain could diversify into distinct sublineages. This phenomenon could be identified for patients enrolled here (13% of them exhibiting a previous urine culture positive to *P. aeruginosa*) and would be interesting to analyze in order to better understand mechanisms of *P. aeruginosa* adaptation to the urinary tract*.* Furthermore, metagenomic studies would be interesting to describe interactions of *P. aeruginosa* with the urinary microbiome^23^.

Our study presents the limitations of a monocentric study. Its results may not be generalized to other geographic areas. Nevertheless, isolates were collected from several clinical wards over a period of 2 years, and the high proportion of singleton STs identified here (72%) was consistent with the *P. aeruginosa* population structure^24^.

In conclusion, this study provides strong evidence of within-host diversity of *P. aeruginosa* urinary isolates, whether phenotypic or genotypic. These findings can complicate diagnosis, treatment and control of *P. aeruginosa* UTI.

## Methods

### *Pseudomonas aeruginosa* strain collection and patient characteristics

From June 2016 to August 2018, we prospectively collected isolates for all monomicrobial urine cultures positive for *P. aeruginosa* (regardless of the level of bacteriuria and leucocyturia) in the Rouen University Hospital. Clinical data were prospectively collected from the hospital’s computerized medical records. The diagnosis of UTI or AB was assigned according to the diagnosis retained by the physician in charge of the patient, and confirmed by a committee made up of microbiologists and infectious disease physicians. A total of 120 patients were consecutively included, 40 with UTI and 80 with AB (Table S4).

For each urine culture, two (in case of low-level bacteriuria, i.e. 2.10^2^ CFU/mL) to five single colonies representative of all morphotypes observed were selected. Bacterial isolates were identified by matrix-assisted laser desorption/ionization time-of-flight (MALDI-TOF) mass spectrometry (Bruker Daltonik GmbH, Bremen, Germany) and stored at − 80 °C for further studies.

### Antibiotic susceptibility

We assessed the activity of 14 antibiotics for the 591 isolates by disk diffusion method on Mueller–Hinton agar (Bio-Rad, Marnes-la-Coquette, France) according to the European Committee on Antimicrobial Susceptibility Testing recommendations (https://www.eucast.org/), as previously described^25^. Antimicrobial disks (Bio-Rad) belonged to distinct antimicrobial groups: penicillins (ticarcillin, ticarcillin-clavulanate, piperacillin, piperacillin-tazobactam), cephalosporins (ceftazidime, cefepime), monobactam (aztreonam), carbapenems (imipenem, meropenem), aminoglycosides (gentamicin, tobramycin, amikacin), and fluoroquinolones (ciprofloxacin, lévofloxacine). For each experiment, a quality control strain (*P. aeruginosa* strain ATCC 27853) was performed. A relative standard deviation of the inhibition zone diameter between intra-sample isolates was calculated for the 15 antibiotics tested.

The proportion of MDR (not susceptible to at least three antimicrobial groups) and XDR (not susceptible to the six antimicrobial groups tested) isolates was determined according to consensus recommendations^26^. The other isolates—resistant to none, one or two groups—were categorized as non-MDR.

Within-host AMR diversity was assessed between intra-sample isolates by comparing AMR profile (non-MDR, MDR, or XDR) and/or according to the relative standard deviation of the inhibition zone diameters within a given profile. Diversity was defined as follows: a major discrepancy as a change to the next category (from non-MDR to MDR or MDR to XDR), while a change from non-MDR to XDR was considered as a very major discrepancy. A minor discrepancy was defined for isolates exhibiting the same AMR profile but with a distinct categorization (susceptible, intermediate or resistant) to one or more antibiotic groups or with a relative standard deviation ≥ 20% for at least one of the 15 antibiotics tested.

To identify clinical risk factors for multidrug resistance (non-MDR versus MDR/XDR), patients exhibiting isolates with different AMR profiles were classified according to the most resistant one (i.e. patients with non-MDR and MDR isolates were classified as MDR patients).

### MLST

MLST was performed for two (in case of low-level bacteriuria) to three isolates per urine culture selected to represent the phenotypic diversity (in terms of morphotype and AMR profile).

MLST was carried out as previously described^27^, except for the use of newly designed primers (Table S5). Briefly, bacterial DNA was extracted by InstaGene Matrix kit (Bio-Rad) according to the manufacturer’s recommendations. PCR products were purified and sequenced by GENEWIZ Europe (Leipzig, Germany). Sequencing data were aligned with BioEdit software (http://www.mbio.ncsu.edu/BioEdit/bioedit.html). Allelic profiles and corresponding sequence types (STs) were assigned using the international PubMLST database (https://pubmlst.org/paeruginosa/). New alleles and STs were submitted to this database. Clinical and microbial data were upload into BioNumerics software (Version 7.6, Applied Maths, Belgium) in order to construct minimum spanning trees based on concatenated sequences.

### Statistical analyzes

The Pearson’s Chi-squared test was used to compare categorical data while ANOVA test was used to analyze continuous data. All tests were two-tailed, and significance was considered at *P* value < 0.05. Statistical analyzes were performed using R software (v.3.5.1).

### Statements on study approvals

We confirm that all methods and protocols used in this study were carried out in accordance with relevant guidelines and regulations. Clinical data and isolates were anonymised so that they were irretrievably unrelated to an identifiable subject. According to French regulation on observational database analyzes, no specific informed consent was required from patients. The study was approved and registered by the Clinical Research and Innovation Delegation of the Rouen University Hospital under number 2018/413/OB. Ethical approval for this study was obtained from the Ethics Committee for Research of the Rouen University Hospital, Rouen, France (No. E2021-77).

## Supplementary Information

Supplementary Information 1.

Supplementary Information 2.
