# Supplementary material for: Phenotypic and genotypic within-host diversity of Pseudomonas aeruginosa urinary isolates
Source: Sci Rep. 2022 Mar 30;12:5421. doi: 10.1038/s41598-022-09234-5 (PMC8967880; doi:10.1038/s41598-022-09234-5)
Supplement: Supplementary file 1 — Supplementary Information 1. [file 41598_2022_9234_MOESM1_ESM.pdf]

Supplementary data

Phenotypic and genotypic within-host diversity of *Pseudomonas aeruginosa* urinary isolates

Table S1. Demographics and clinical features for the 120 patients with *P. aeruginosa* bacteriuria assessed as urinary tract infection (UTI) or asymptomatic bacteriuria (AB)

Characteristics	Entire cohort (n=120)	UTI (n=40)	AB (n=80)	*P*
**Demographics** *no.* (%)				
Age (years) [min;max]	64 [0;101]	62 [0;101]	65 [1;96]	0.55
Male gender	77 (64%)	31 (78%)	46 (58%)	**0.031**
**Clinical features** *no.* (%)				
Urinary tract catheter	60 (50%)	21 (53%)	39 (49%)	0.70
Bacteraemia caused by *P. aeruginosa*	9/60 (15%)	8/27 (30%)	1/33 (3%)	**<0.005**
**Comorbid diseases** *no.* (%)				
Urinary comorbidities	61 (51%)	23 (58%)	38 (48%)	0.30
Benign prostatic hyperplasia	25/77 (32%)	7/31 (23%)	18/46 (39%)	0.15
Neurogenic bladder	12 (10%)	3 (8%)	9 (11%)	0.52
Genitourinary abnormality	9 (8%)	5 (13%)	4 (5%)	0.14
Urinary diversion	9 (8%)	3 (8%)	6 (8%)	1
Kidney transplant	8 (7%)	4 (10%)	4 (5%)	0.30
Nephrolithiasis	6 (5%)	4 (10%)	2 (3%)	0.076
**Charlson index** (avg±SD)*	5.4 ± 2.9	5.0 ± 2.4	5.6 ± 3.1	0.26
Myocardial infarct	2 (2%)	0 (0%)	2 (3%)	NA
Congestive heart failure	11 (10%)	6 (16%)	5 (7%)	0.10
Peripheral vascular disease	14 (12%)	5 (14%)	9 (12%)	0.78
Cerebrovascular disease	20 (18%)	6 (16%)	14 (18%)	0.80
Dementia	24 (21%)	7 (19%)	17 (22%)	0.70
Chronic pulmonary disease	28 (25%)	6 (16%)	22 (29%)	0.15
Connective tissue disease	15 (13%)	3 (8%)	12 (16%)	0.27
Ulcer disease	5 (4%)	3 (8%)	2 (3%)	0.18
Mild liver disease	5 (4%)	1 (3%)	4 (5%)	0.54
Uncomplicated diabetes	22 (19%)	12 (32%)	10 (13%)	**0.014**
Diabetes mellitus with end organ damage	12 (11%)	3 (8%)	9 (12%)	0.56
Hemiplegia	15 (13%)	5 (14%)	10 (13%)	0.94
Moderate or severe renal disease	25 (22%)	5 (14%)	20 (26%)	0.13
Localized tumor	29 (25%)	9 (24%)	20 (26%)	0.85
Metastatic solid tumor	4 (4%)	0 (0%)	4 (5%)	NA
Leukemia	2 (2%)	1 (3%)	1 (1%)	0.59
**Other comorbidities**	12 (10%)	4 (10%)	8 (10%)	1
Non-genitourinary transplant	2 (2%)	2 (5%)	0 (0%)	NA
Immunocompromised conditions [a]	10 (8%)	2 (5%)	8 (10%)	0.35
**Clinical wards** *no.* (%)				
Medicine	46 (38%)	14 (35%)	32 (40%)	ND
Surgery	32 (27%)	12 (30%)	20 (25%)	ND
Intensive care	22 (18%)	8 (20%)	14 (18%)	ND
Rehabilitation care	13 (11%)	3 (8%)	10 (13%)	ND
Pediatry	7 (6%)	3 (8%)	4 (5%)	ND
**Clinical histories within 6 months** *no.* (%)				
Previous urine culture positive to *P. aeruginosa*	15 (13%)	4 (10%)	11 (14%)	0.56
Previous antibiotic treatment	93 (78%)	34 (85%)	59 (74%)	0.16
Previous hospitalization	103 (86%)	35 (88%)	68 (85%)	0.71
Previous urinary tract manipulation	82 (68%)	27 (68%)	55 (69%)	0.89
Including previous urological surgery	21 (18%)	9 (23%)	12 (15%)	0.31

*Charslon index was measured only for adult cases (*i.e.* 114 patients, 37 with UTI, and 77 with AB)

[a] Immunocompromised conditions referred to diseases not included in the Charlson index (AIDS, lymphoma, leukemia) and excluded post-transplant immunosuppressor treatment (specifically classified into renal transplant or non-genitourinary transplant)

min: minimum; max: maximum; P: *P-value*; NA : not applicable ; ND ; not determined

Table S2. Clinical risk factors associated with multidrug resistant or extensively drug resistant *P. aeruginosa* urinary isolates

Characteristics	AMR profiles		
	Non-MDR (*n*=87)	MDR/XDR (*n*=33)	*P*
**Demographics** *no. (%)*			
Age (years) [min;max]	65 [0;101]	62 [22;95]	0.43
Male gender	56 (64%)	21 (64%)	0.94
**Clinical features** *no. (%)*			
Urinary tract infection	28 (32%)	12 (36%)	0.66
Urinary tract catheter	39 (45%)	21 (64%)	0.066
Bacteraemia caused by *P. aeruginosa*	8/41 (20%)	1/19 (5%)	0.15
**Comorbid diseases** *no. (%)*			
Urinary comorbidity	43 (49%)	18 (55%)	0.62
Charlson index (avg±SD)*	5.6 ± 2.9	4.94 ± 2.8	0.24
Other comorbidity	7 (8%)	5 (15%)	0.25
**Clinical histories within the previous 6 months** *no. (%)*			
Previous urine culture positive to *P. aeruginosa*	6 (7%)	9 (27%)	**<0.005**
Previous antibiotic treatment	61 (70%)	32 (97%)	**<0.005**
Penicillins	49 (56%)	23 (70%)	0.21
Cephalosporins	26 (30%)	17 (52%)	**0.034**
Carbapenems	6 (7%)	6 (18%)	0.088
Aminoglycosides	14 (16%)	11 (33%)	**0.047**
Quinolones	9 (10%)	13 (39%)	**<0.001**
Other antimicrobials	30 (34%)	22 (67%)	**<0.005**
Previous hospitalization	74 (85%)	29 (88%)	0.69
Previous urinary tract manipulation	57 (66%)	25 (76%)	0.28
Including previous urological surgery	15 (17%)	6 (18%)	0.90

*Charslon index was measured only for adult cases (*i.e.* 114 patients, 37 with UTI, and 77 with AB)

MDR: multidrug resistant; XDR: extensively drug resistant; AMR: antimicrobial resistance

min: minimum; max: maximum; SD: standard deviation; *P: P-value*

Table S3. Sequence types (STs) identified by multilocus sequence typing for the 358 *P. aeruginosa* isolates

STs	No. of patients	No. of isolates
395	11	33
308	11	32
253	7	20
309	6	18
235	6	17
446	5	13
244, 298	4	12
111, 313, 389	3	9
27	3	8
207	3	5
17, 560, 1248	2	6
683	2	5
3233*	2	4
132, 170, 175, 232, 233, 234, 252, 254, 258, 261, 274, 291, 316, 319, 379, 381, 386, 390, 527, 532, 633, 676, 701, 769, 1028, 1076, 1125, 1270, 2125, 2128, 2438, 2445, 2554, 2683, 2859, 3225*, 3226*, 3227*, 3228*, 3229*, 3230*, 3231*, 3234*	1	3
483, 2406	1	2
3232*	1	1

Ten new STs (*) were incorporated in the online database (https://pubmlst.org/paeruginosa/), three of them being defined by new alleles (for *aroE*, *mutL*, and *ppsA*).

Table S5. Primers used for multilocus sequence typing

Locus	Primers	Sequences (5'–3')	References
acsA	acsA-F_[A]	ACCTGGTGTACGCCTCGCTGAC	Curran *et al.* 2004
	acsA-R_[A]	GACATAGATGCCCTGCCCCTTGAT	Curran *et al.* 2004
	acsA-F_[S]	CGACTACCGTCCGGGCGAAGT	This study
	acsA_R_[S]	TCATGGTCGCCGAACAGGGTC	This study
aroE	aroE-F_[A]	TGGGGCTATGACTGGAAACC	Curran *et al.* 2004
	aroE-R_[A]	TAACCCGGTTTTGTGATTCCTACA	Curran *et al.* 2004
	aroE-F_[S]	TGGTCTATGACGCGCAACTGG	This study
	aroE-R_[S]	CAACTGTCGGCGCAGCGTCTC	This study
guaA	guaA-F_[A]	CGGCCTCGACGTGTGGATGA	Curran *et al.* 2004
	guaA-R_[A]	GAACGCCTGGCTGGTCTTGTGGTA	Curran *et al.* 2004
	guaA-F_[S]	CCCGGAAGTCACCCACACCAA	This study
	guaA-R_[S]	CCCAGCTCCAGGCCGATCTTG	This study
mutL	mutL-F_[A]	GGTAGCGCGCCTGACCATGAC	This study
	mutL-R_[AS]	GCCGGACTCTCCAGCACGCTC	This study
	mutL-F_[S]	ACCGAGGGCCGCGACATGCAG	This study
nuoD	nuoD-F_[A]	CGCCACCGAGTTCGATCCCTACT	This study
	nuoD-R_[A]	GATACCCTTGGTCGCCTCGATCAT	This study
	nuoD-F_[S]	TCCCTGTCCGCCGCCAAGCAG	This study
	nuoD-R_[S]	GGAACTCGCGGACCAGCTTGT	This study
ppsA	ppsA-F_[A]	GGTCGCTCGGTCAAGGTAGTGG	Curran *et al.* 2004
	ppsA-R_[A]	GGGTTCTCTTCTTCCGGCTCGTAG	Curran *et al.* 2004
	ppsA-F_[S]	GGCCGCCCGATGGACATCGAA	This study
	ppsA-R_[S]	GGATTGCCGACGTTCATCATG	This study
trpE	trpE-F_[A]	GCGGCCCAGGGTCGTGAG	Curran *et al.* 2004
	trpE-R_[AS]	CCCGGCGCTTGTTGATGGTT	Curran *et al.* 2004
	trpE-F_[S]	CGCAGCGCATGTCCATCGAAT	This study

F: Forward primer; R: Reverse primer; [A]: primer for PCR amplification; [S]: primer for Sanger sequencing
